# Coccolithophore calcification response to past ocean acidification and climate change

**DOI:** 10.1038/ncomms6363

**Published:** 2014-11-17

**Authors:** Sarah A. O’Dea, Samantha J. Gibbs, Paul R. Bown, Jeremy R. Young, Alex J. Poulton, Cherry Newsam, Paul A. Wilson

**Affiliations:** 1 Department of Ocean and Earth Science, National Oceanography Centre, Southampton, University of Southampton, Waterfront Campus, European Way, Southampton SO14 3ZH, UK; 2 Department of Earth Sciences, University College London, Gower Street, London WC1E 6BT, UK; 3 Ocean Biogeochemistry and Ecosystems, National Oceanography Centre, Southampton, Waterfront Campus, Southampton SO14 3ZH, UK

## Abstract

Anthropogenic carbon dioxide emissions are forcing rapid ocean chemistry changes and causing ocean acidification (OA), which is of particular significance for calcifying organisms, including planktonic coccolithophores. Detailed analysis of coccolithophore skeletons enables comparison of calcite production in modern and fossil cells in order to investigate biomineralization response of ancient coccolithophores to climate change. Here we show that the two dominant coccolithophore taxa across the Paleocene–Eocene Thermal Maximum (PETM) OA global warming event (~56 million years ago) exhibited morphological response to environmental change and both showed reduced calcification rates. However, only *Coccolithus pelagicus* exhibits a transient thinning of coccoliths, immediately before the PETM, that may have been OA-induced. Changing coccolith thickness may affect calcite production more significantly in the dominant modern species *Emiliania huxleyi*, but, overall, these PETM records indicate that the environmental factors that govern taxonomic composition and growth rate will most strongly influence coccolithophore calcification response to anthropogenic change.

## Abstract (web summary)

Calcifying organisms such as planktonic coccolithophores may be particularly vulnerable to increased ocean acidification. Here, O’Dea *et al.* show that two fossil coccolithophore species exhibited reduced calcification rates during a global warming acidification event 56 million years ago.

## Graphical abstract

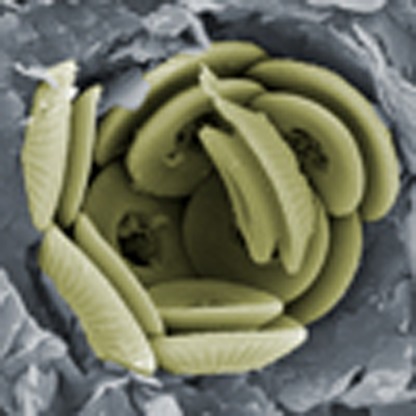

Acidification and reduced carbonate saturation of the oceans are measureable responses to anthropogenic emissions of carbon dioxide into the atmosphere^1^. As major pelagic producers of CaCO_3_ in the modern ocean, the sensitivity of coccolithophores (single-celled phytoplankton) to changes in surface water chemistry is of particular relevance for ocean biogeochemical cycles and climate feedback systems (for example, ref. 2). Coccolithophores build exoskeletons from individual CaCO_3_ (calcite) plates—coccoliths—that cover the cell surface and form a protective barrier (the coccosphere). Our current understanding of coccolithophore responses to ocean acidification (OA) is predominantly based on calcification rate experiments (coccolith calcite production per cell, per unit time), which indicate complex, often species- or strain-specific, impacts (for example, refs 3, 4, 5). There is also experimental evidence that coccolithophore species have the ability to evolve and adapt to the acidifiying carbonate chemistry conditions that are projected for the future, over the relatively short timescales of multiple generations (100–1,000 s of generations)^6^. The geological record of fossil coccolithophores is remarkably complete (stratigraphically and taxonomically) throughout the past 220 million years^7^, and therefore provides a valuable means by which to test hypotheses of coccolithophore response both to long-term environmental change and abrupt climate perturbations. However, interrogation of the geological record for the specific impact of OA on coccolithophore calcification is challenging, because calcification rate cannot readily be determined in fossil populations, and therefore direct comparison with responses measured in modern culture and field experiments is inhibited.

At a basic level, the fossil remains of coccolithophores provide a means by which to estimate coccosphere calcite quotas (the mass of calcite associated with individual coccospheres or cells), by combining the physical parameters of individual coccoliths (that is, size, thickness and mass)^8^,^9^,^10^ with the cell geometry of intact coccospheres (that is, the size and number of coccoliths per cell)^11^. However, coccosphere calcite quotas cannot be directly used to estimate calcification rate, because high coccosphere calcite quotas can be associated with low calcification rates if the production of coccoliths and/or rate of cell division is low and *vice versa*. We can overcome this problem by combining measurements of coccosphere calcite quotas (including a newly revised method for calculating coccolith thickness) with estimates of cell division rates, utilizing a recently developed method of coccosphere geometry analysis^11^. This approach enables a closer comparison of fossil and modern species, because it focuses on biomineralization at a cellular level. This technique is underpinned by recent observations of modern *Coccolithus pelagicus* batch culture experiments, supported by field data, which reveal a systematic relationship between coccosphere size (external diameter, *Ø*), coccolith length (*C*_*L*_) and the number of coccoliths per coccosphere (*C*_*N*_)^11^. Importantly, variation in *C*_*N*_ is linked to the growth phase of the cell, such that small cells with few coccoliths are produced during exponential growth phase (normal, rapid division) and larger cells with more coccoliths are produced during early stationary phase (slowed cell division).

Here, we perform a suite of morphometric measurements on fossil coccospheres and their coccoliths to test for skeletal changes across the Paleocene–Eocene Thermal Maximum (PETM), a prominent global carbon cycle perturbation that occurred ~56 million years ago. The PETM is associated with a carbon isotope excursion (CIE) to lower values, global mean surface water warming of ~4–5 °C and widespread deep water acidification^12^,^13^. Proxy records of surface water pH change also suggest that variations in surface water chemistry accompanied deep water acidification at the CIE^14^. While the absolute magnitudes of surface water chemistry change remain poorly constrained, modelling studies suggest that the PETM might have been associated with a ~1.5-unit decline in carbonate saturation or a 0.1–0.45-unit decline in pH^15^,^16^,^17^. In order to enable the study of a greater range of fossil and modern coccolithophore taxa, which can differ greatly in size and crystallography, we have modified existing methods to measure coccolith thickness (for example, refs 8 and 10). The new technique provides an estimate of the amount of calcite per coccolith for ancient taxa, here specifically *C. pelagicus* and *Toweius pertusus*, which were volumetrically and numerically dominant during the late Paleocene and early Eocene (for example, refs 18, 19, 20). In addition, we address the issue of fossil preservation, one of the major problems encountered in geological materials, by utilizing exquisite hemipelagic fossils from palaeo-shelf-sea sediments of New Jersey (Bass River), supplemented by material from California (Lodo Gulch) and Tanzania Drilling Project Site 14 (refs 21, 22), which provide the best-preserved materials available for the PETM. Our morphometric analyses show that calcification rate declined in *T. pertusus* and *C. pelagicus* populations following the CIE onset, and provide evidence for species-specific adaptive response to environmental change during the PETM interval. Furthermore, the thinning of *C. pelagicus* coccoliths immediately before the CIE indicates an additional biomineralization response, likely caused by a different environmental control, possibly OA.

## Results

### Coccolith morphometric measurements and coccosphere geometry

**Figure 1:**
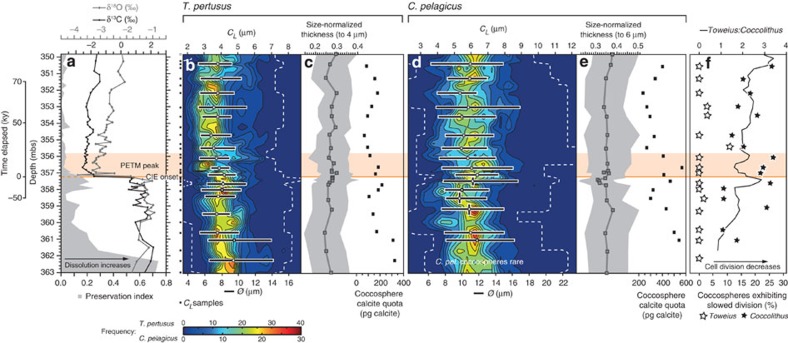
Stable isotope, coccolith preservation and morphometric records at Bass River, New Jersey. (**a**) Bulk carbon (black squares) and bulk oxygen isotopes (grey diamonds; data from John *et al.*^21^) with a quantitative nannofossil preservation index (data from Gibbs *et al.*^38^; grey shading). Time from the initial carbon isotope excursion (CIE) is indicated (kyrs; following John *et al.*^21^), as are depths from which coccolith length (*C*_*L*_) measurements were collected (black squares). (**b**,**d**) Frequency data for coccolith lengths (*C*_*L*_; totalling 3,200 and 3,050 measurements for *T. pertusus* and *C. pelagicus*, respectively) are interpolated to equal depth steps of 10 cm, with minimum and maximum size-bins (dashed line). Mean coccosphere diameters (*Ø*; white squares) with 5th and 95th percentiles of each population (horizontal black bars) and the sampling interval (vertical black bars) are shown, calculated from a total of 507 and 375 coccospheres for *Toweius* and *Coccolithus*, respectively. (**c**,**e**) Mean coccolith size-normalized thickness for *T. pertusus* and *C. pelagicus* (dark grey squares), with the 5th and 95th percentiles of each sample (grey shading). Uncertainty on mean size-normalized thickness is calculated as two s.d.s across the bootstrap results at the length to which thickness is being normalized and does not exceed ±0.008 μm. Mean coccosphere calcite quotas are shown (black squares). (**f**) The percentage of each population that exhibits coccosphere geometry typical of slowed cell division (early stationary growth phase, see ref. 11; open stars for *T. pertusus* and closed stars for *C. pelagicus*) and the ratio of *T. pertusus* to *C. pelagicus* coccoliths (black line). The onset of the CIE (orange line) and interval of peak warmth during the PETM (orange shading) are indicated (following John *et al.*^21^).

**Figure 2:**
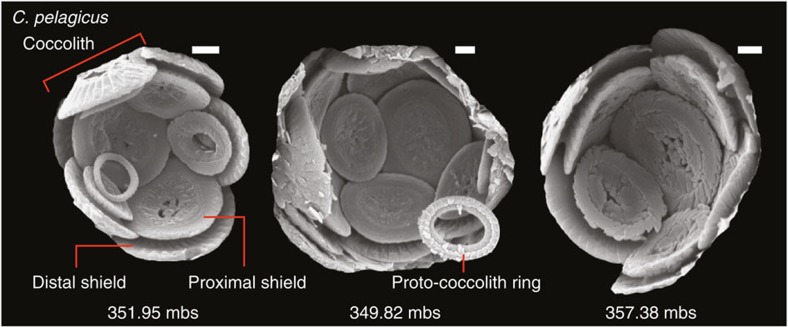
Scanning electron micrographs of *C. pelagicus* at Bass River, New Jersey. Sample depths are 351.95, 349.82 and 357.38 mbs as indicated. The minimum in size-normalized thickness of *C. pelagicus* occurs between 357.38 and 357.36 mbs. Individual scale bars indicate 1 μm.

Our morphometric data reveal a clear, positive relationship between coccolith size (distal shield length, *C*_*L*_) and coccolith thickness (partial thickness of the proximal shield, *C*_*T*_; see Methods section) at all sites, which indicates that the thickness of coccoliths typically increases in proportion with their size (Supplementary Figs 1 and 2b,c,g,h), in effect an allometric relationship. To critically assess the variations in *C*_*T*_ that are beyond this relationship, we calculate size-normalized coccolith thickness (see Methods section). Calculating size-normalized thickness provides a quantitative estimate of the divergence between the *C*_*L*_ and *C*_*T*_ records, and therefore describes the variation in *C*_*T*_ that is independent of the ‘normal’ *C*_*L*_ and *C*_*T*_ relationship (Supplementary Fig. 2d,i). Our high-resolution downcore records at Bass River demonstrate species-specific differences in size-normalized thickness, with a notable minimum in mean size-normalized thickness of *Coccolithus pelagicus* at ~357.56 m below surface (mbs; Fig. 1), immediately before the onset of the CIE. Variations in size-normalized thickness are decoupled from coccolith preservation, with no evidence of coccolith thinning at levels of increased dissolution (Fig. 1), where a secondary modification of *C*_*T*_ would be most likely. This observation is further supported by scanning electron microscope (SEM) images, which show no preservation-related coccolith thinning that could impact our thickness estimates (Fig. 2 and Supplementary Methods).

Modal *C*_*L*_ values indicate a step-shift decrease in *Toweius pertusus* across the peak of the PETM (centred on 356.83 mbs; Fig. 1b) from values typically greater than ~4 μm to values <3.8 μm. For *C. pelagicus*, downcore modal *C*_*L*_ values reveal minor fluctuations and a transient increase during the peak of the PETM, and a longer-term increase in maximum values (Fig. 1d). Coccosphere geometry (the relationship between *C*_*L*_, *Ø* and *C*_*N*_) is more tightly constrained in *T. pertusus* than in *C. pelagicus*, which shows a broader range of *Ø* and *C*_*L*_ and a highly variable *C*_*N*_ (consistent with ref. 11 and Supplementary Fig. 3). The downcore record of coccosphere *Ø* broadly tracks variations in modal *C*_*L*_ values for both taxa (Fig. 1b,d), with divergence occurring where *C*_*N*_ varies. Using the proportion of coccosphere population that exhibits stationary phase geometry, with non-dividing coccospheres identified as those that have *C*_*N*≥16_ for *C. pelagicus* and *C*_*N*≥12_ for *T. pertusus*^11^, we are able to estimate the general growth phase of our individual populations through time across the PETM interval. *Toweius* populations typically display exponential phase coccosphere geometry (characterized by a low proportion of coccospheres with *C*_*N*≥12_), thereby indicating that high levels of cell division are maintained across the PETM (Fig. 1f). *Coccolithus* coccosphere geometries, however, reveal intervals of slowed division, characterized by an increased proportion of large coccospheres with *C*_*N*≥16_, (and a reduced proportion of post-division coccospheres with *C*_*N≤*8_), particularly across the onset and peak of the PETM (consistent with ref. 11; Fig. 1f). These levels of reduced cell division in *C. pelagicus* are broadly supported by transient increases in the relative numbers of disarticulated *T. pertusus* to *C. pelagicus* coccoliths preserved in the sediments (Fig. 1f).

### Coccosphere calcite quotas and calcification rates

**Figure 3:**
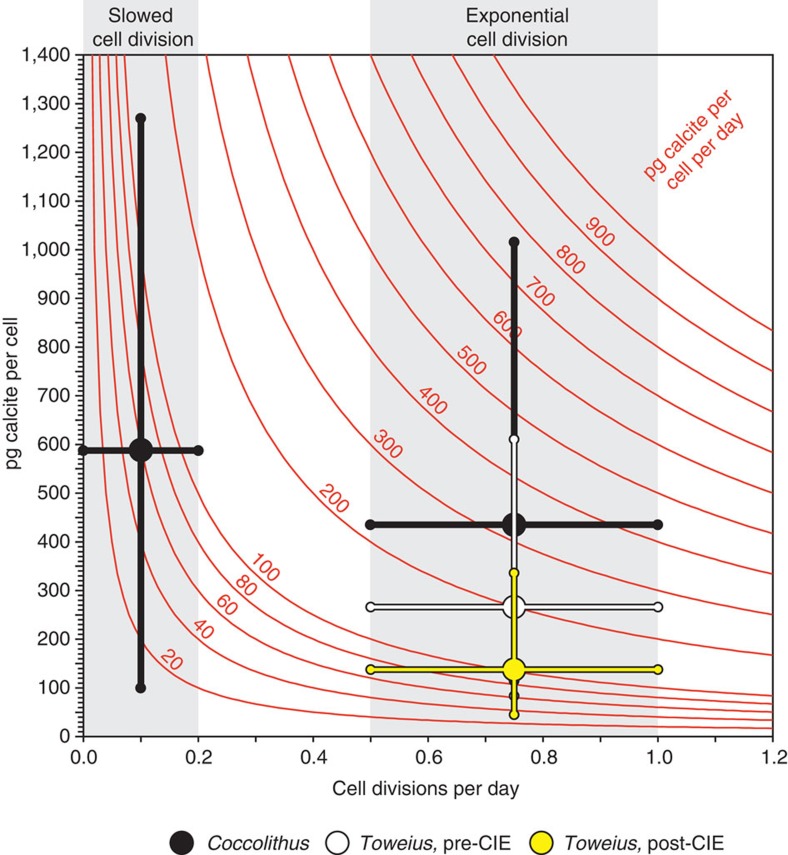
Estimating calcification rate for fossil coccolithophore species. Calcification rates (curved red lines) are derived by multiplying the amount of calcite produced per cell by the number of cell divisions per day. Mean (large circles) and the 5th and 95th percentiles (vertical lines) of coccosphere calcite quotas are shown (*Toweius* in white and yellow for pre- and post-CIE onset populations, respectively; *Coccolithus* in black). Populations exhibiting exponential phase coccosphere geometry (normal rates of cell division) are plotted between 0.5 and 1.0 divisions per day and likely vary within this range. *Coccolithus* populations that are exhibiting early stationary phase coccosphere geometry (slowed division; characterized by a high proportion of *C*_*N≥16*_) across the onset and peak of the PETM are plotted between 0 and 0.2 divisions per day. Small circles indicate intersections between calcification rates and either the measured percentile range of coccosphere calcite quotas (on vertical lines) or the inferred range of divisions per day (on horizontal lines).

We use *C*_*L*_ measurements to calculate coccolith mass by applying species-specific shape factors (following ref. 23; see Methods section). We then adjust these shape factor calculations by using the change in our mean size-normalized thickness measurements to provide estimates of variation in coccolith mass that might result directly from the thinning and thickening of coccoliths (see Methods section; Supplementary Fig. 2e,j). We find that the net impact of thickness variation on coccolith mass across the PETM accounts for up to ~5–11% and ~6–16% of the amount of calcite per coccolith for *Toweius pertusus* and *Coccolithus pelagicus*, respectively (see Methods section). This change in calcite mass per coccolith is modest in comparison with variations in coccosphere calcite quotas that result from the observed changes in *C*_*L,*_ *Ø* and *C*_*N*_ across the PETM, which are up to 500% for *T. pertusus* (that is, up to a fivefold difference in mean coccosphere calcite mass across the record) and 240% for *C. pelagicus* (Fig. 1c,e). Although these variations in coccosphere calcite quota are significant, they do not constitute evidence for a change in the coccolithophore calcification rate during the PETM, because they do not account for potential variations in coccolith production or cell division. Using a similar range of cell division rates to those observed in *C. pelagicus* and *Emiliania huxleyi* culture and field experiments^11^ (0.5–1.0 and 0–0.2 divisions per day for exponential and stationary phase, respectively; see Methods section), we conservatively estimate that for *Coccolithus*, the observed shift to early stationary phase cell division could result in at least a near halving of the calcification rate of those populations, from 220–440 to <120 pg calcite per cell per day, despite the increase in maximum coccosphere calcite quotas during the peak of the PETM (Figs 1e and 3). For *Toweius* populations, which maintain high levels of cell division across the PETM, the effect of variation in coccolith production and cell division on calcification rate is less marked. However, unlike *Coccolithus* populations, the step-shift decrease in *Toweius C*_*L*_ and *Ø* values at ~356.83 mbs, and the resultant decrease in coccosphere calcite quota from pre- to post-CIE onset populations (Fig. 1b,c), have a clear influence on calcification rate. Based on the ranges of coccosphere calcite quota for pre- and post-CIE onset populations, and a realistic range of growth rate variation within exponential phase (0.5–1.0 divisions per day), this change in morphology results in a reduction of calcification rate in *Toweius* populations from 140–260 to 70–130 pg calcite per cell per day, again, an approximate halving of calcification rate (Fig. 3; for reference, field populations of modern *E. huxleyi* typically produce calcite at a rate of 25–75 pg calcite per cell per day)^24^.

## Discussion

Our records of coccolith size-normalized thickness and coccosphere geometry at Bass River have enabled us to identify three key diagnostic features of coccolithophore calcification across the PETM. First, the minor reduction in *Coccolithus pelagicus* size-normalized thickness immediately before the CIE; second, the significant decrease in calcite production by *Coccolithus* populations during the interval of peak warmth; and third, the decrease in calcite production in *Toweius* populations post-CIE onset (Figs 1 and 3). *Coccolithus* calcite production was likely controlled by changes in growth phase, resulting from the overall environmental changes associated with the PETM^11^, with similar growth phase variations also evidenced by low-resolution time series data from other sites including Lodo Gulch (California), the Bay of Biscay and Tanzania^11^. However, the decrease in calcite production in *Toweius* populations reflects a reduction in size of coccospheres and associated coccoliths via a population shift towards smaller cells, likely resulting from either a phenotypic shift or biogeographic introduction, rather than a change in overall cell division. Our combined records of coccosphere calcite quota, *C*_*L*_, *Ø* and *C*_*N*_, therefore indicate that the significant changes in calcification rate of the *Coccolithus* and *Toweius* populations reflect responses that are species specific, involving reduced cell division in *Coccolithus* and a prolonged cell (and coccolith) size decrease in *Toweius*. Importantly, the reduced calcite production is likely linked with the environmental factors (predominantly temperature, nutrient availability and irradiance) that we know have major influence on growth phase, cell size^11^,^25^,^26^ and species biogeography^27^ in the natural environment, and is unlikely to predominantly result from OA in either species.

**Figure 4:**
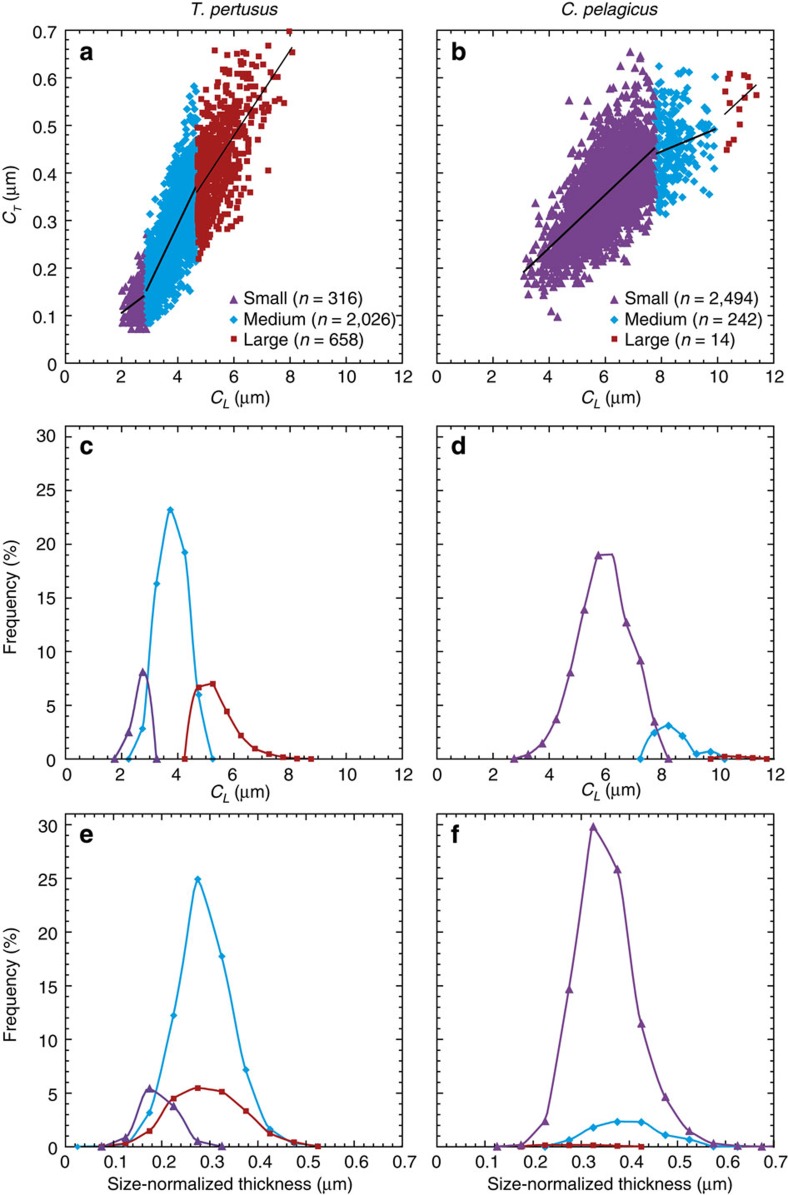
Species morphotypes at New Jersey, California and Tanzania. (**a**,**b**) Coccolith length (*C*_*L*_) and thickness (*C*_*T*_) measurements for size-defined morphotypes of *T. pertusus* and *C. pelagicus*, identified using mixture analyses. Frequency distributions of *C*_*L*_ (**c**,**d**) and size-normalized thickness (**e**,**f**) of each size-defined morphotype. For *T. pertusus* ‘small’ is <2.9 μm, ‘medium’ is >2.9 to <4.7 μm and ‘large’ is >4.7 μm, whereas for *C. pelagicus*, ‘small’ is <7.7 μm, ‘medium’ is >7.7 to <9.9 μm and ‘large’ is >9.9 μm.

The reduction in *C. pelagicus* size-normalized thickness differs from the coccosphere geometry data (*C*_*L*_, *Ø* and *C*_*N*_) as it occurred before the onset of the CIE and over a short and discrete time interval. As changes in *C. pelagicus* size-normalized thickness are apparently decoupled from mean cell size and the ecophysiological factors (primarily growth phase) that govern coccosphere geometry^11^, the thinning was most likely caused by a different environmental factor, with OA being a primary candidate. Although changing coccolith thickness is the type of biomineralization response we might expect to result from ocean chemistry changes as biomineralization becomes more metabolically expensive, coccolith thinning is still not equivocal evidence for OA and the underlying mechanism for how a thinning response might occur requires cautious consideration. The observed variation in coccolith size-normalized thickness could be owing to, first, a biomineralization response to changes in surface water chemistry with phenotypic plasticity in *C*_*T*_ within successive ancestor-descendent populations, and/or, second, selection of genotypically distinct populations with subtle differences in *C*_*T*_. A degree of phenotypic plasticity that accounts for the relatively minor changes in mean size-normalized thickness is certainly plausible. However, as internal coccolithogenesis occurs within a pH-regulated vacuole^28^, the selection of genotypes with different coccolith thicknesses may represent a more realistic explanation. As a first approximation, mixture analyses (see Methods section) performed on *C*_*L*_ data from all sites support this hypothesis, as they indicate the presence of at least three discrete, size-defined morphotypes of each fossil ‘species’ of *T. pertusus* and *C. pelagicus*, (Fig. 4a–d and Supplementary Table 1); the rare and smallest morphotype of *T. pertusus* has a slightly different characteristic size-normalized thickness (Fig. 4e and Supplementary Fig. 4). This morphotype diversity is likely a considerable underestimate, because genetic and ecological studies of living coccolithophores show that genotypic diversity within species or subspecies is significantly higher than is suggested by the subtle morphological differences seen in coccoliths and coccospheres (for example, refs 29, 30, 31). In addition, broad genotypic diversity has recently been documented in experiments with modern *Emiliania huxleyi*, revealed as subtle differences in the physiology and ecological niche of individual strains, such that changes in environment result in selection of different genotypes^6^.

Whether a function of phenotypic plasticity or selection of genotypically distinct populations, if the thinning of *C. pelagicus* coccoliths is linked to changes in surface water carbonate chemistry, this would suggest that OA preceded the onset of the CIE at Bass River by ~3,000–4,000 years (based on the Bass River age model that correlates the CIE to orbitally tuned data from Ocean Drilling Program Site 690 (refs 21, 32)). Although the CIE provides for reliable identification of the PETM in the geological record, a range of other chemical and biotic evidence indicate that significant environmental change preceded the CIE at multiple sites^32^. The thinning of *C. pelagicus* coccoliths is an addition to this existing evidence for pre-CIE changes, which includes TEX_86_ palaeothermometry data that indicate substantial sea surface temperature increase and an anomalous abundance acme of the subtropical dinoflagellate cyst *Apectodinium*, at least along the New Jersey margin and in the North Sea^32^ (Supplementary Fig. 4a). The significant biotic response seen in two key plankton groups, coccolithophores and dinoflagellates, indicates rapid pre-CIE environmental change, because the short generation time of these organisms typically increases their resilience to all but the most abrupt environmental perturbation. While the cause of pre-CIE environmental change remains unclear, evidence for pre-CIE surface water OA indicates carbon cycle involvement. However, any precursor increase in atmospheric carbon dioxide would need to be from a different source to that of the isotopically light carbon, which caused the main CIE, in order to not significantly affect the carbon isotope record^32^,^33^. Currently, it is not clear what the source of this carbon might be as, while volcanic outgassing is an obvious candidate, it is currently difficult to reconcile timescales of addition of known carbon sources with the rapid environmental changes necessary to induce the observed range of biotic features^32^.

Overall, our PETM data suggest that changes in the environmental factors that affect coccolithophore growth (that is, temperature, nutrient availability and irradiance) had the potential to significantly influence global pelagic carbonate production in the Paleogene, and this is likely to be just as significant today. A change in division rate in modern *Coccolithus* populations could actually have a greater influence on overall calcite production than that observed during the PETM, because the modern *C. pelagicus* subspecies, *pelagicus* and *braarudii*, typically produce larger coccoliths than Paleogene *C. pelagicus*^11^. This is reflected in estimates of mean rate of calcite production during exponential division, which are ~535 and 3,360 pg per cell per day for modern *C. pelagicus* ssp. *pelagicus* and ssp. *braarudii*, respectively, compared with ~332 pg per cell per day for Paleogene populations (modern data obtained from ref. 11). However, global calcite production is also dependent on species biogeography and abundance, and while modern *C. pelagicus* is a dominant and large coccolithophore similar to its fossil counterparts, its biogeography is more restricted in the modern ocean (for example, refs 34 and 35). Instead, modern coccolithophore populations are typically dominated by smaller and more lightly calcified taxa, the most abundant species being *E. huxleyi,* a descendent of *Toweius*. Shifts in *E. huxleyi C*_*L*_ and *Ø*, similar to those we have documented in *Toweius* during the PETM, could therefore have significant impact on modern rates of global calcification. Moreover, coccolith thickness variations may play a greater role today than in the past because *E. huxleyi* has an unusually variable, perforate coccolith architecture that results in up to fourfold differences in calcite mass, for very similar sized coccoliths, across its morphotypes^23^. This perforate architecture could potentially accommodate much greater levels of thickness and mass change than we observed in the dominant Paleogene coccoliths, in response to the more rapid OA that is predicted for the coming centuries. Ultimately, though, it is the factors that govern the taxonomic composition of coccolithophore communities, their biogeography, growth rate and adaptive response, which will likely exert the most significant control on overall calcite production by coccolithophores, with comparatively little direct impact on biomineralization from changes in ocean chemistry.

## Methods

### Material and site descriptions

Our morphometric data are from coccoliths and intact coccospheres from the palaeo-shelf-sea sediments of Bass River, New Jersey (39°36.42 N, 74°26.12 W), supplemented by material from Lodo Gulch, California (36°32.18 N, 120°38.48 W) and Tanzania Drilling Project Site 14 (9°1659.89 S, 39°30 45.04 E). The Paleogene sections at Bass River, Lodo Gulch and Tanzania are all stratigraphically expanded with average sedimentation rates of ~10, ~24.6 and 4 cm per kyr, respectively^21^,^20^. The Bass River section is stratigraphically complete for the first ~100 kyr of the 170 kyr CIE^32^. All three sections comprise clay-rich fine sandy and silty sediments deposited above the lysocline in outer shelf to upper slope environments^20^,^21^,^22^, thereby minimizing the effects of secondary dissolution on the preserved coccoliths^21^,^20^. Preservation was assessed using SEM images, taken from sediment surfaces following techniques used in ref. 36.

### Sampling strategy and morphometric measurements

All measurements were made using standard smear slides, with samples spanning the PETM section at Bass River. The highest resolution sampling of 6 cm intervals was undertaken where carbon isotope values show the greatest stratigraphic variability. We have measured morphometric parameters from statistically significant samples of 100 disarticulated coccoliths per species. Measurements were collected from the first 100 coccoliths of each species identified per slide and include coccolith size (distal shield length, *C*_*L*_) and coccolith thickness (*C*_*T*_, to quantify partial thickness of the proximal coccolith shield according to its birefringence under cross-polarized light; see Supplementary Methods). We calculate size-normalized thickness using the mean slope of a bootstrapped linear regression between *C*_*L*_ and *C*_*T*_ for each sample, including 100 measurements that are selected at random from the data set for 100,000 iterations in the bootstrap. To determine the distribution of size-normalized thickness for each sample, we effectively collapse the data to a consistent, species-specific *C*_*L*_ (4 μm for *T. pertusus* and 6 μm for *C. pelagicus*) along the mean slope of the bootstrapped regression (Supplementary Fig. 5), as follows:


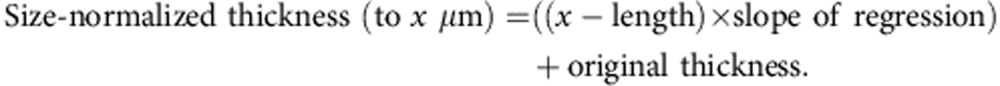


In addition, we present the first high-resolution coccosphere size record for the PETM, with measurements of up to 30 fossil coccospheres per species per sample where available, totalling 507 coccospheres for *T. pertusus* and 375 for *C. pelagicus* from 23 and 24 samples, respectively. Morphometric fossil coccosphere data include coccosphere size (external diameter *Ø*) and the number (*C*_*N*_) and size (*C*_*L*_) of intact coccoliths on each coccosphere (following ref. 11). All measurements were collected using Cell^D imaging software, with images taken using a colour DP71 video camera attached to an Olympus BX51 cross-polarizing microscope.

### Estimating coccosphere calcite quotas

We estimate coccosphere calcite quotas for intact coccospheres by estimating the mass of each coccolith and multiplying this by *C*_*N*_. Coccolith mass is estimated as:


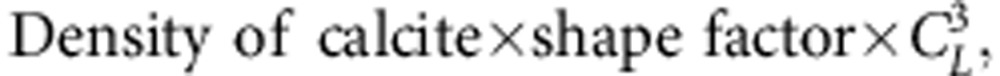


following ref. 23, using 2.7 as the density of calcite, 0.060 as a shape factor for *C. pelagicus* and a conservative placolith shape factor of 0.055 for *T. pertusus*. We also include an estimate of *C*_*T*_ variations using our measurements from disarticulated coccoliths, volumetrically modifying the shape factors by adding the difference between mean size-normalized thickness and the expected thickness of the same-sized coccolith, based on its shape factor. We include minimum and maximum values for the estimated net impact of *C*_*T*_ change on coccolith carbonate mass, with minimum values accounting for a change in *C*_*T*_ that is restricted to the proximal shield, while maximum values account for a change in *C*_*T*_ that alters the proximal shield, distal shield and tube cycle.

### Estimating calcification rates for fossil populations

Our estimated fossil coccolithophore calcification rates assume that rates of exponential phase cell division during the Paleogene were similar to those observed in culture and field experiments of modern *C. pelagicus* and *E. huxleyi* (typically between ~0.5 and 1 divisions per day) and that populations displaying early stationary phase (slowed division) coccosphere geometries are dividing slower than minimum exponential rates (<0.2 divisions per day)^11^. These division rates likely represent realistic but conservative rates for Paleogene communities, which lived in warmer waters than modern taxa and may therefore have undergone slightly higher rates of division. Fossil coccolithophore calcification rates are derived by multiplying the amount of calcite produced per cell by the number of cell divisions per day.

### Using mixture analyses to identify size-defined morphotypes

We provide a first order approximation of the presence of different species morphotypes by performing mixture analyses on *C*_*L*_ data from all sites using Palaeontological Statistics. Mixture analyses aim to identify the parameters of normally distributed groups within a pooled sample, and use a maximum likelihood approach to assign each *C*_*L*_ measurement to one group. Here, mixture analyses identify up to three groups, or ‘morphotypes’, within each species. Results are based on a single morphometric parameter, *C*_*L*_, and therefore do not provide definitive evidence of full morphotypic variability, but instead indicate a conservative number of possible differences within a species. We calculate the Akaike Information Criterion (AIC_c_) values for each mixture analysis (that is, identifying 1, 2 or 3 morphotypes of each species; Supplementary Table 1) using Palaeontological Statistics, and convert these to Akaike weights between 0 and 1, following ref. 37. Akaike weights can be interpreted as an approximate probability (between 0 and 1) that each of the mixture analysis scenarios tested (that is, 1, 2, 3 or more morphotypes) is the best candidate. The Akaike weights sum to one across the candidate scenarios. For both species, the mixture analysis that defines three morphotypes records the lowest AIC_c_ value and the Akaike weight that is nearest to 1 (1.0 and 0.95 for *Toweius* and *Coccolithus*, respectively; Supplementary Table 1).

## Author contributions

S.J.G. conceived the project. The research was designed by S.A.O and S.J.G., with advice from P.R.B. and J.R.Y. S.A.O. collected the data, with contributions from S.J.G. and C.N. P.R.B. performed SEM imaging. S.A.O., S.J.G. and P.R.B. interpreted findings and wrote the paper, with advice from J.R.Y., A.J.P. and P.A.W.

## Additional information

**How to cite this article:** O’Dea, S. A. *et al.* Coccolithophore calcification response to past ocean acidification and climate change. *Nat. Commun.* 5:5363 doi: 10.1038/ncomms6363 (2014).

## Supplementary Material

Supplementary Information Supplementary Figures 1-7, Supplementary Table 1, Supplementary Methods and Supplementary References.
