# Supplementary material for: Coccolithophore calcification response to past ocean acidification and climate change
Source: Nat Commun. 2014 Nov 17;5:5363. doi: 10.1038/ncomms6363 (PMC4243242; doi:10.1038/ncomms6363)
Supplement: Supplementary Information — Supplementary Figures 1-7, Supplementary Table 1, Supplementary Methods and Supplementary References. [file ncomms6363-s1.pdf]

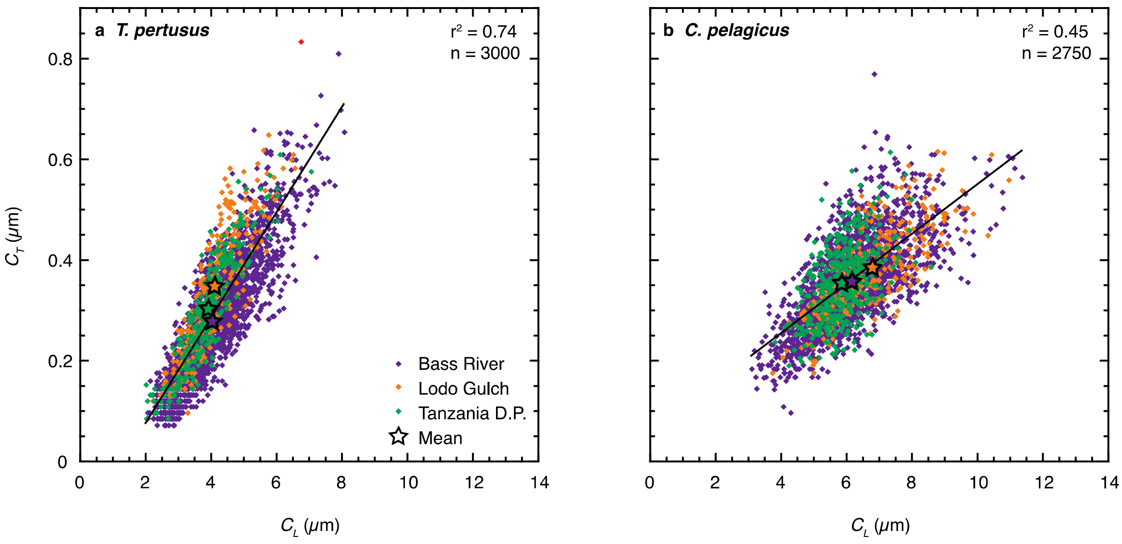

Supplementary Figure 1. Coccolith length (C_L) and thickness (C_T) at Bass River, Lodo Gulch and Tanzania.

a, *Toweius pertusus* and **b**, *Coccolithus pelagicus* at Bass River (purple), Lodo Gulch (orange) and Tanzania (green). Stars indicate site means.

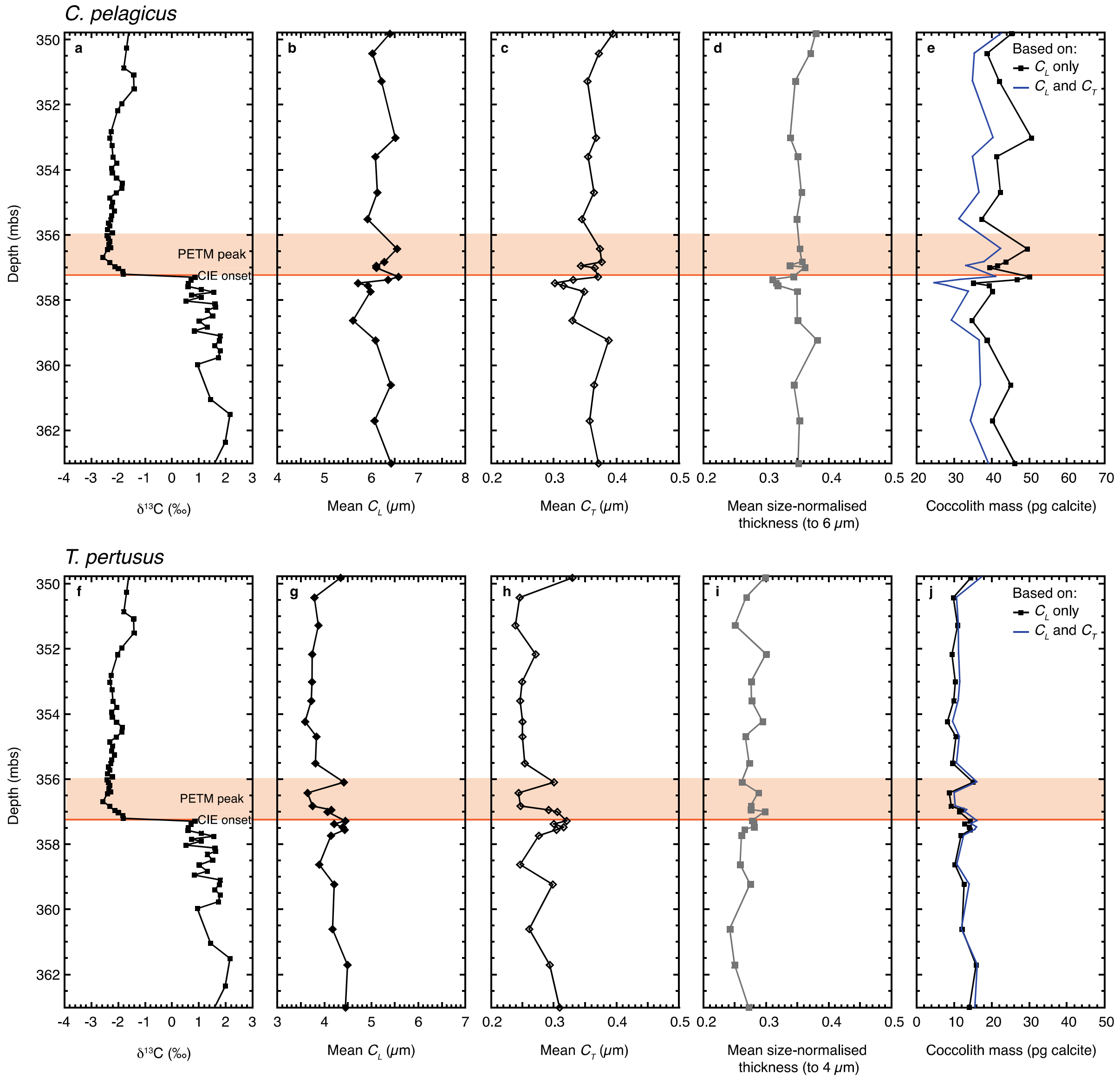

Supplementary Figure 2. Downcore stable isotope and coccolith morphometric records at Bass River, New Jersey.

a, **f**, Bulk carbon isotope values (data from John *et al.*[1]) are shown. **b**-**e** and **g-j** show mean coccolith length (C_L), coccolith thickness (C_T), size-normalised thickness and coccolith mass estimates for *C. pelagicus* and *T. pertusus*, respectively. Coccolith mass estimates based on variations in C_L are shown (black squares) along with coccolith mass estimates that include variations in C_L and C_T, which assume that change in C_T alters the proximal shield, distal shield and tube cycle (blue line). Variations in thickness account for a larger proportion of coccolith mass change in *C. pelagicus* than *T.pertusus.* The onset of the carbon isotope excursion (CIE; orange line) and interval of peak warmth during the PETM (orange shading) are indicated (following John *et al.*[1]).

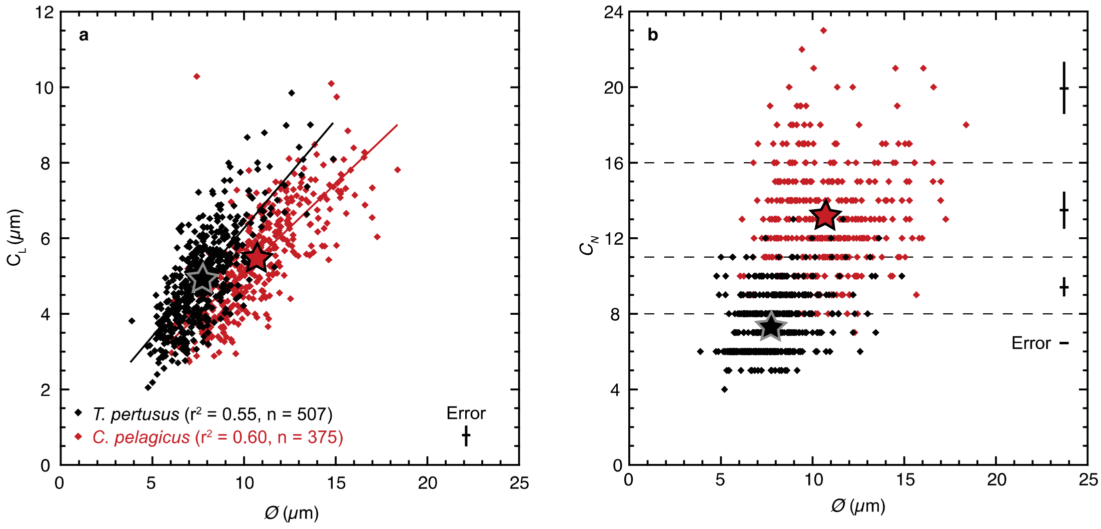

Supplementary Figure 3. Coccosphere morphometric data for *T. pertusus* and *C. pelagicus* at Bass River

a, Coccolith length (C_L) and coccosphere size (*Ø*). **b**, Number of coccoliths per coccosphere (C_N) and *Ø*. Stars indicate means. *Ø* error is based on measurement replicates and C_L error is based on replicates and uncertainty in coccolith tube cycle to coccolith diameter conversion (see Fig. 2 of Gibbs *et al.*[2]). C_N uncertainty is minimal when coccospheres have 8 or fewer coccoliths, but increases with increasing C_N (indicated by dashed lines).

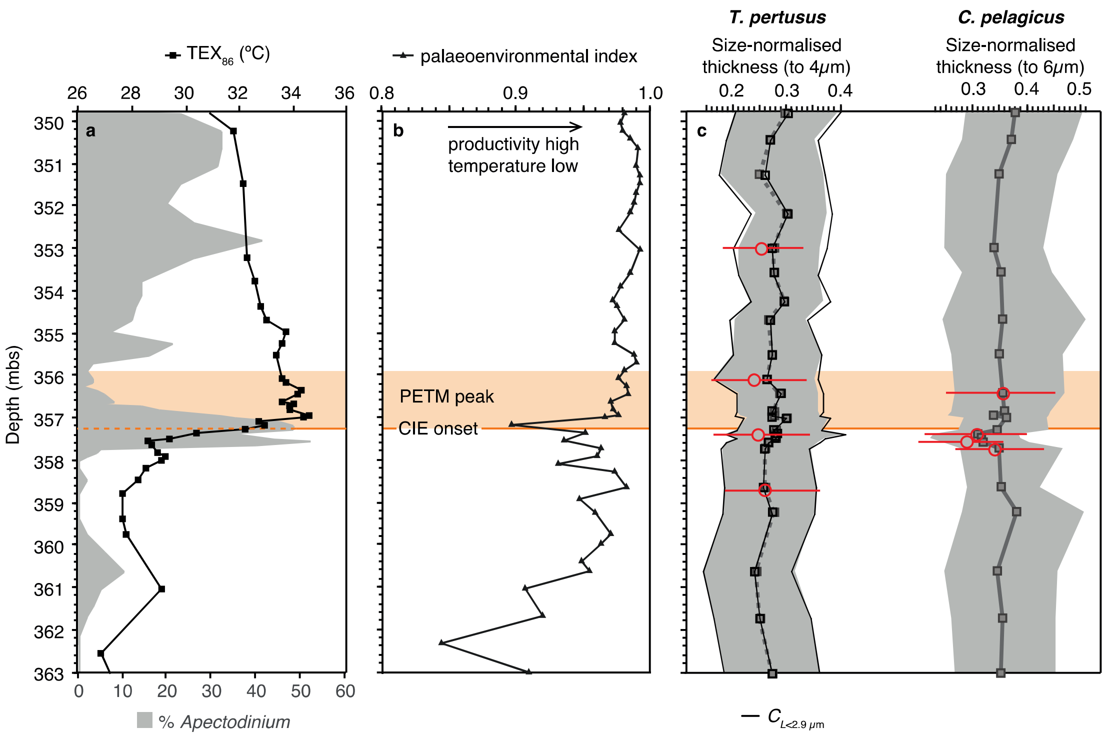

Supplementary Figure 4. Temperature, palaeoenvironment and coccolith size-normalised thickness records at Bass River, New Jersey.

a, TEX_{86} temperature and *Apectodinium* percent abundance records are from Sluijs *et al.*[3], with the TEX_{86} temperature scale based on the calibration of Schouten *et al.*[4]. **b**, Nannofossil palaeoenvironmental index from Gibbs *et al.*[5]. **c**, Mean size-normalised thickness (as in Fig. 1) for *T. pertusus* and *C. pelagicus*, with the mean and 5th and 95th percentiles of repeated samples indicated (red circles). Mean size-normalised thickness and 5th and 95th percentiles of *T. pertusus* calculated without the small ($C_{L<2.9\,\mu m}$) morphotype (black squares and lines) are superimposed on the original pattern (as in Fig. 1, here shown with a dashed line and grey shading). The carbon isotope excursion (CIE; orange line) and interval of peak warmth during the PETM (orange shading) are indicated (following John *et al.*[1]).

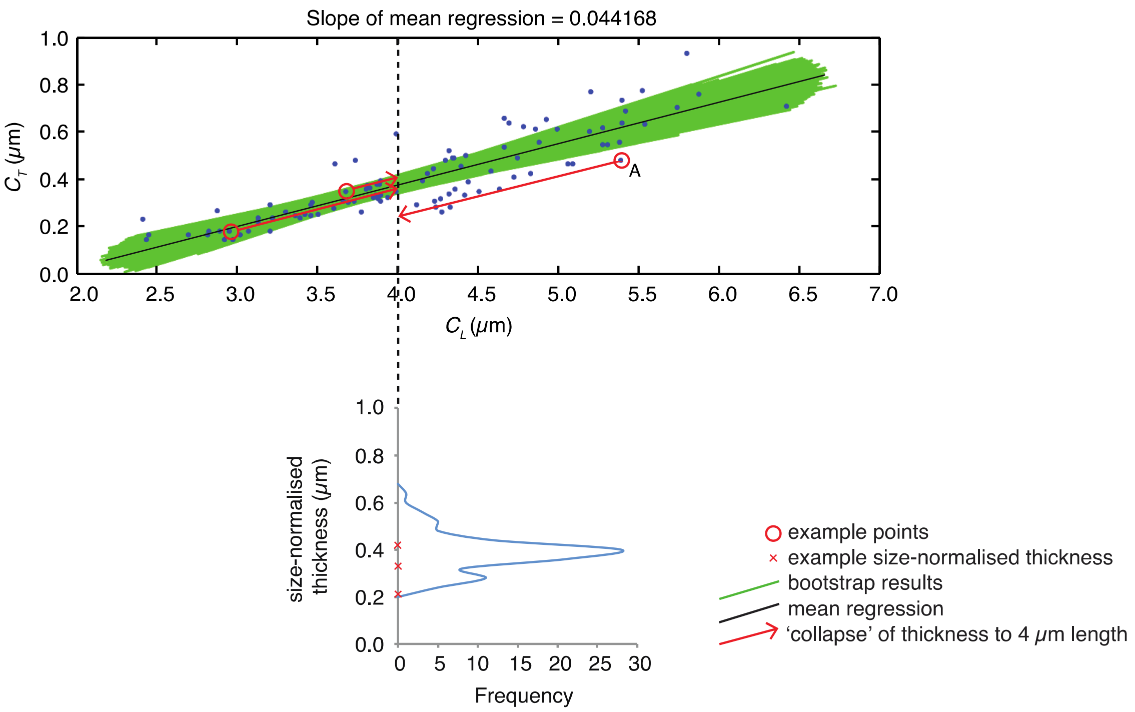

Supplementary Figure 5. Calculating coccolith size-normalised thickness from length and thickness measurements.

Results of the bootstrapped linear regression for 100 measurements from 1 sample of coccolith length (C_L) and thickness (C_T) of *T. pertusus* at Bass River (blue points). 100,000 bootstrap results are shown (green lines), with the mean regression superimposed upon these (black). Red arrows describe the 'collapse' of three example data points (circled red) to a consistent length (4 µm) along the mean slope of the bootstrapped regression. Size-normalised thickness for point 'A' (length = 5.39 µm; thickness = 0.48 µm) is calculated as ((4 – 5.39) x 0.044168) + 0.48 = 0.42 µm. The histogram shows the distribution for all 100 coccoliths in this sample, with size-normalised thickness for the three example data points indicated (red cross).

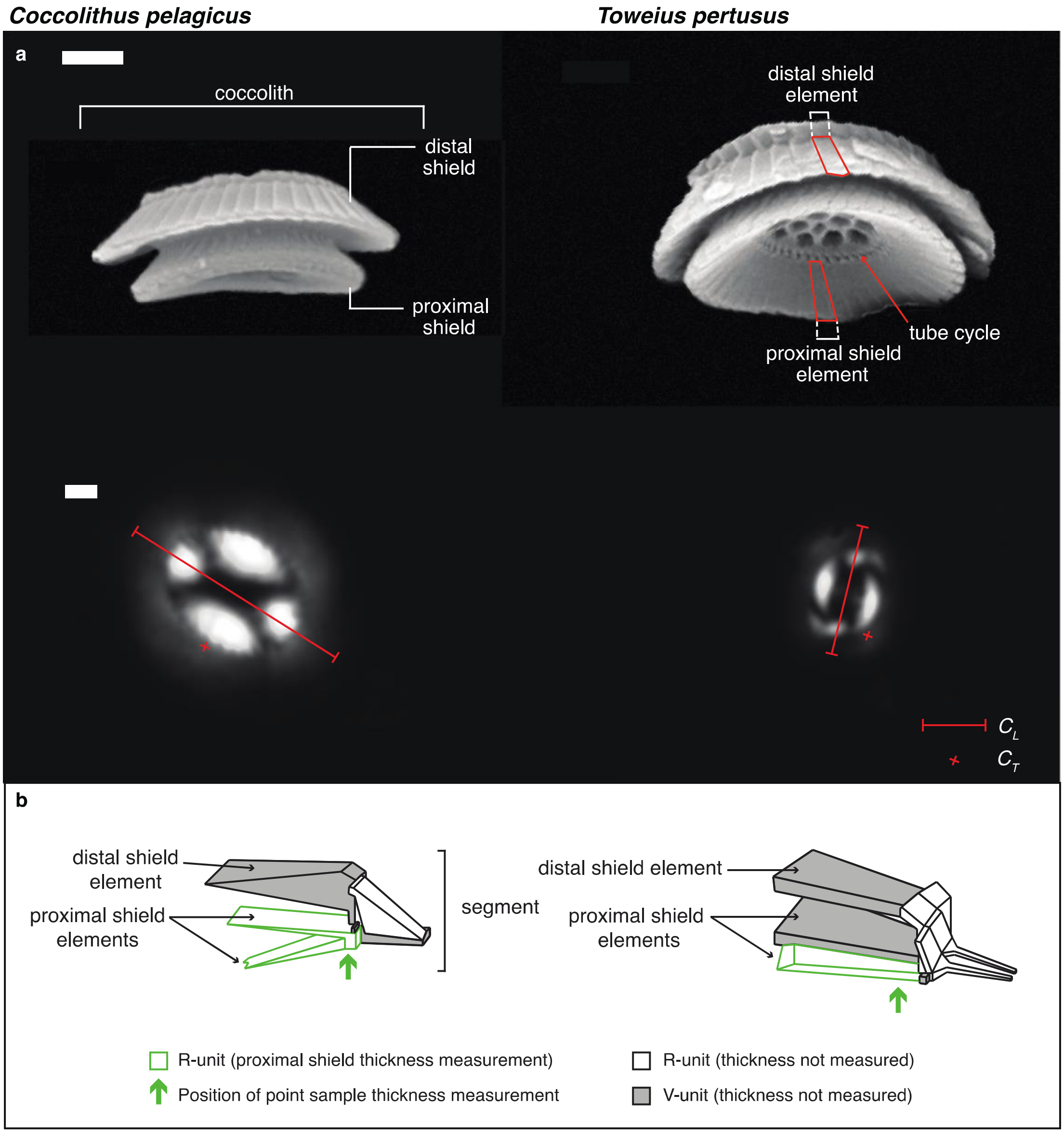

Supplementary Figure 6. Coccolith structure and crystallographic axes.

a, Scanning electron micrographs and light micrographs of *C. pelagicus* and *T. pertusus* coccoliths. Scale bars indicate 1 µm. **b**, Schematic diagrams of coccolith structure and crystallographic axes of V- and R-unit elements for *C. pelagicus* and *T. pertusus* (redrawn after Young *et al.*[6]) with the position of point sample thickness measurements indicated (green arrows).

[Following page] Supplementary Figure 7. Calibration of grey value to calcite thickness.

a, First principles theoretical $\sin^2$ relationship between greyscale values, retardation and thickness of calcite crystals. **b**, True natural greyscale spectra measured at equal distances (arbitrary units) along a quartz wedge at different microscope light intensities. The theoretical $\sin^2$ relationship between grey value and retardation has been fitted for each of the natural spectra (blue shading). The natural spectrum produced with the 'pre-set' microscope and camera conditions utilised for coccolith thickness measurements is shown with orange diamonds, and has been fitted with a first principles theoretical curve (red). The section of the calibration used to determine coccolith thickness herein is marked (dashed red line). Grey values from 0 to ~6 arbitrary units along the quartz wedge record a decrease in thickness to 0 µm, which results from the processes used to manufacture the quartz wedge. Thickness of calcite where the radial c-axis is inclined at 10º from horizontal (e.g., in the proximal shield of *C. pelagicus*) is indicated.

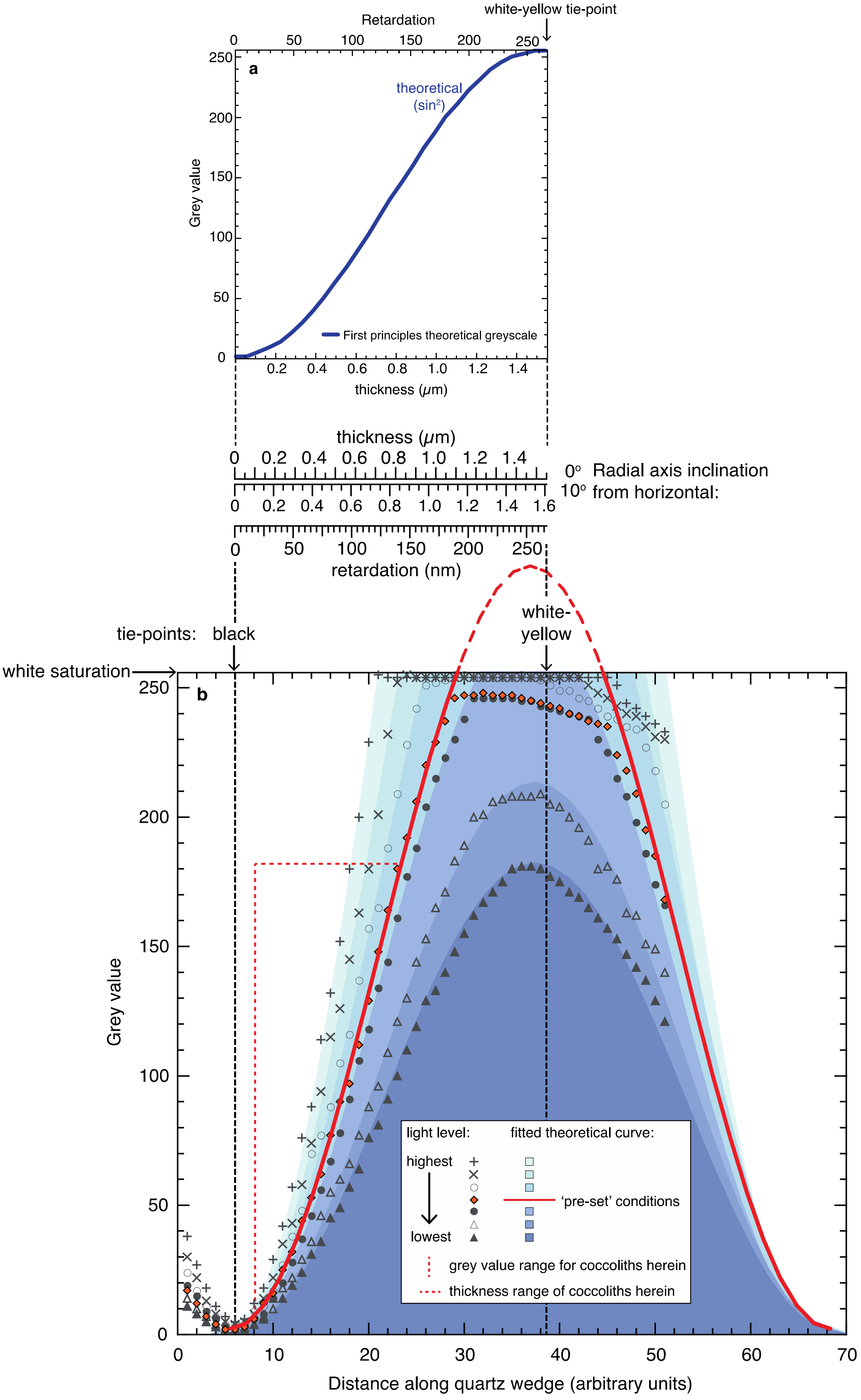
Retardation
white-yellow tie-point
0 50 100 150 200 250
a
theoretical (sin²)
Grey value
First principles theoretical greyscale
0.2 0.4 0.6 0.8 1.0 1.2 1.4
thickness (μm)
thickness (μm)
0° Radial axis inclination
10° from horizontal:
retardation (nm)
tie-points: black
white-yellow
white saturation
b
Grey value
light level:
fitted theoretical curve:
highest
lowest
'pre-set' conditions
grey value range for coccoliths herein
thickness range of coccoliths herein
Distance along quartz wedge (arbitrary units)

Supplementary Table 1. Akaike Information Criterion for mixture analyses

	Coccolithus			*Toweius*		
**No. of morphotypes modelled**	1	2	3	1	2	3
**Akaike Information Criterion (AIC_c)**	3575	3432	3426	2722	2506	2458
**Akaike weight**	0.000	0.047	0.953	0.000	0.000	1.000

Supplementary Methods

Measuring the thickness of Paleogene coccoliths

Methods that utilise birefringence to estimate the mass of calcite associated with individual coccoliths make use of the relationship between the thickness of a calcite crystal and its interference (or polarisation) colour when viewed under cross- or circular-polarised light[7,8]. When passing through a calcite crystal, polarised light is refracted into two perpendicular rays that travel at different velocities, with the slower ray being retarded in relation to the faster ray. The degree of retardation is a function of birefringence (the difference between the refractive indices) and the thickness of the crystal. Viewed under circular-polarised light, or with a consistent orientation of the crystallographic-axes (c-axes) under cross-polarised light, calcite crystals reveal interference colours, the first order (retardation values of 0-550 nm) of which range from black (zero order), through grey to white, white-yellow, orange and red, with increasing crystal thickness.

Coccoliths comprise individual calcite crystal units with c-axes of either vertical to sub-vertical (V-units) or horizontal to sub-horizontal (radial, R-units) orientation[9,10]. When coccoliths are in plan view (their typical orientation in standard smear slides), the V-unit elements will be dark under cross- or circular-polarised light because a vertical c-axis does not produce any retardation, therefore only those elements with radial c-axes are clearly visible. Existing techniques estimate the weight of individual coccoliths by measuring birefringence of R-units under cross- or circular-polarised light. The techniques utilise the relationship between interference colour and calcite

crystal thickness to calibrate from grey pixel values (obtained from black and white images) to coccolith thickness, integrating this across the coccolith area to estimate weight[11-17]. These techniques have been applied to the skeletal remains of modern and Quaternary coccolithophores but are unsuitable for use on pre-Quaternary coccoliths for two reasons. First, unlike the coccoliths of Quaternary and modern Isochrysidales taxa, which are predominantly composed of R-unit elements, many pre-Quaternary coccoliths incorporate large V-unit crystals and are therefore not fully birefringent across the coccolith (Supplementary Fig. 6). Second, the dominant Paleogene placolith taxa are substantially larger than modern and Quaternary coccoliths, and their thickness produces birefringence that extends beyond the grey part of the spectrum of interference colours. We have therefore modified existing methods to measure the point of maximum thickness of R-unit elements of the proximal shield, which, for our focal taxa *Coccolithus* and *Toweius*, remain within the grey-white part of the spectrum of interference colours (Supplementary Fig. 6). Although the tube cycles of *Coccolithus* and *Toweius* also incorporate R-units (Supplementary Fig. 6), tube cycle thickness often exceeds 1.55 µm, thereby producing interference colours that extend beyond the grey-white part of the spectrum, which cannot be measured accurately using our technique. Specifically, we measure the greyscale value of the point of maximum birefringence (and therefore thickness) of the proximal shield when the tube cycle is in focus.

Coccolith thickness calibration

Conversion of point sample grey values to calcite thickness requires, first, an understanding of the relationship between greyscale values and calcite thickness and second, application of this relationship to specific microscope and camera systems. First principles theoretical calculations, supported by experimental data, indicate that a $\sin^2$ relationship exists between microscope light intensity and retardation of birefringent materials[18,19]. Therefore, provided that the relationship between microscope light intensity and camera-assigned greyscale is linear, the relationship between grey value and calcite thickness (which is proportional to retardation), is also, theoretically,

described by a $\sin^2$ curve (Supplementary Fig. 7a). Our observations across various natural spectra, including quartz wedges (the thickness of which increases linearly with distance along the wedge), mica grains and stacked birefringent plastic sheets confirm the $\sin^2$ shape of the relationship between camera-recorded grey values and crystal thickness (Supplementary Fig. 7b). Importantly, greyscale values measured at equal distances (referred to as arbitrary units) along a quartz wedge provide a means by which to identify black (zero order) and the white-yellow transition in interference colour, i.e., 0 and 267 nm retardations, respectively. These provide tie-points to corresponding theoretical calcite thicknesses of 0 and 1.55 µm, respectively (Michel Lévy chart; Supplementary Fig. 7b), which can be used to convert distance along the quartz wedge to calcite thickness.

Ideally, conversion of point sample grey values to calcite thickness would utilise a microscope and camera set-up that reproduces the first principles ($\sin^2$) theoretical curve between grey value and thickness in its optimum position, i.e., ‘pinned’ to minimum and maximum greyscale values such that black = 0 and white = 255 and these correspond exactly with thicknesses of 0 and ~1.55 µm, respectively (Supplementary Fig. 7a). In practice, however, it is difficult to reproduce this ‘optimal’ relationship between grey value and crystal thickness by altering the microscope and camera conditions (primarily those factors that influence light intensity: lamp illumination, condenser alignment, position of polarisers, magnification and camera exposure time). For simplicity, and reliability of reproducibility tests, all coccolith thickness measurements herein are taken at a consistent light intensity, controlled by using a pre-set lamp illumination (manufacturer-defined) and a constant alignment of the condenser, position of polarisers, magnification (x100 objective) and camera exposure time (all checked prior to data collection for each sample). At these specific ‘pre-set’ microscope conditions, grey values collected at equal distances along a quartz wedge reveal that the natural spectrum saturates prematurely (i.e., saturation occurs where thickness = ~1.1 µm, rather than 1.55 µm). Therefore, to constrain the position of the pre-set (saturated) greyscale curve with respect to the first principles ($\sin^2$) theoretical curve, we collected greyscale values from

multiple natural spectra along a quartz wedge at a range of light levels, both higher and lower than the pre-set light intensity (Supplementary Fig. 7b). From this, we assign a thickness value to each of the grey values, as predicted by this theoretical relationship. These thickness values can be adjusted to account for the inclination of the radial c-axis from horizontal in coccoliths, which is negligible in *T. pertusus* but ~ 10º in *C. pelagicus*[7] (Supplementary Fig. 7).

The greyscale saturation produced using the pre-set microscope and camera conditions does not affect our ability to measure the thickness of Paleogene *Coccolithus* and *Toweius* specimens, because the grey values collected herein do not exceed 185, and therefore remain significantly below white saturation values of the applied theoretical curve. However, the premature greyscale saturation of the pre-set microscope conditions might become a consideration when measuring other taxa or other time intervals, particularly if specimens are thicker than those measured herein, requiring a necessary adjustment of the microscope light intensity or camera exposure time. Also of note are the subtle differences in the shape of the natural spectra and their theoretical counterparts where grey values exceed ~245, a function of the difference between theoretical greyscale values and the conversion of natural interference colours to greyscale (Supplementary Fig. 7b).

Uncertainty and reproducibility of size-normalised thickness measurements

Uncertainty on mean size-normalised thickness is estimated for each sample as two standard deviations across the bootstrap results at the length to which thickness is being normalised. To minimise this uncertainty, we estimate size-normalised thickness at the length that shows least uncertainty from a bootstrap of all samples for each species, which is 4 µm for *T. pertusus* and 6 µm for *C. pelagicus*. Measurement error is quantified based on replicates for both species and is incorporated into the bootstrapped regression by assigning error to each coccolith measurement, randomly selected from within 2 standard deviations of calculated measurement error.
Reproducibility of the size-normalised thickness data was assessed by repeating measurements of length and size-normalised thickness for 4 samples for each species (Supplementary Fig. 4c). We

note that the repeated estimates of size-normalised thickness differ more from the original samples in *T. pertusus* than in *C. pelagicus*, probably due to the greater variability in size-normalised thickness in *T. pertusus* populations. Importantly, the minimum in size-normalised thickness recorded in the original samples of *C. pelagicus* is also evident from the repeated measurements.

Preservational control on coccolith thickness

Coccolith preservation is exceptionally good at Bass River, Lodo Gulch and Tanzania across the PETM, revealing only minor dissolution from organic carbon degradation (degraded samples were avoided where possible) with no evidence of secondary overgrowth. Breakage and minor dissolution of the radial c-axis units of the proximal shield revealed by SEM images (Figure 2) does not affect our thickness measurements, which consistently record maximum point thickness of the proximal shield. Where we do observe slightly poorer preservation, it is mostly manifested as incised edges, breakage and loss of the R-units of the proximal shield, without resulting in obvious thinning that could impact our estimates of coccolith thickness.

Supplementary References

1. John, C. M. *et al.* North American continental margin records of the Paleocene-Eocene thermal maximum: Implications for global carbon and hydrological cycling. *Paleoceanography* **23,** doi:10.1029/2007pa001465 (2008).

2. Gibbs, S.J. *et al*. Species-specific growth response of coccolithophores to Palaeocene-Eocene environmental change. *Nature Geoscience,* **6**, 218-222 doi:10.1038/ngeo1719 (2013).

3. Sluijs, A. *et al*. Environmental precursors to rapid light carbon injection at the Palaeocene/Eocene boundary. *Nature* **450,** 1218-1222, doi:10.1038/Nature06400 (2007).

4. Schouten, S. *et al*. Extremely high sea-surface temperatures at low latitudes during the middle Cretaceous as revealed by archaeal membrane lipids. *Geology* **31,** 1069-1072 (2003).

5. Gibbs, S. J., Stoll, H. M., Bown, P. R. & Bralower, T. J. Ocean acidification and surface water carbonate production across the Paleocene-Eocene thermal maximum. *Earth and Planetary Science Letters* **295,** 583-592, doi:10.1016/J.Epsl.2010.04.044 (2010).

6. Young, J. R., Davis, S. A., Bown, P. R. & Mann, S. Coccolith ultrastructure and biomineralisation. *Journal of Structural Biology* **126,** 195-215 (1999).

7. Young, J. R. Description and analysis of coccolith structure. In, Hamrsmid, B. and Young, J.R., (eds.) *Proceedings of the 4th International Nannoplankton Association Conference Prague 1991.* 35-71, Knihovnicka zemniho plynu a nafta, Hodonin (1993).

8. Higgins, M.D. Imaging birefringent minerals without extinction using circularly polarized light. *The Canadian Mineralogist* **48**, 231-235, doi:10.3749/canmin.48.1.231 (2010).

9. Young, J. R., Didymus, J. M., Bown, P. R., Prins, B. & Mann, S. Crystal assembly and phylogenetic evolution in heterococcoliths. *Nature* **356,** 516-518 (1992).

10. Young, J.R. *et al*. Guidelines for coccolith and calcareous nannofossil terminology, *Paleontology* **40** 875-912 (1997).

11. Beaufort, L. Weight estimates of coccoliths using the optical properties (birefringence) of calcite. *Micropaleontology* **51,** 289-297 (2005).

12. Beaufort, L., Probert, I. & Buchet, N. Effects of acidification and primary production on coccolith weight: Implications for carbonate transfer from the surface to the deep ocean. *Geochemistry Geophysics Geosystems* **8,** doi:10.1029/2006gc001493 (2007).

13. Beaufort, L., Couapel, M., Buchet, N., Claustre, H. & Goyet, C. Calcite production by coccolithophores in the south east Pacific Ocean. *Biogeosciences* **5,** 1101-1117 (2008).

14. Beaufort, L. *et al*. Sensitivity of coccolithophores to carbonate chemistry and ocean acidification. *Nature* **476,** 80-83, doi:10.1038/Nature10295 (2011).

15. Cubillos, J., Henderiks, J., Beaufort, L., Howard, W. R. & Hallegraeff, G. M. Reconstructing calcification in ancient coccolithophores: Individual coccolith weight and morphology of *Coccolithus pelagicus* (sensu lato). *Marine Micropaleontology* **92-93,** 29-39 (2012).

16. Bollmann, J. Technical note: weight approximation of single coccoliths inferred from retardation estimates using a light microscope equipped with a circular polariser – (the CPR Method). *Biogeosciences Discussions,* **10**, 11155-111179, doi: 10.5194/bgd-10-11155-2013 (2013).

17. Fuertes, M.A., Flores, J.A., & Sierro, F.J. A new technique for observing calcareous nannofossils: methodology and applications. *14th INA Conference, Reston, Virginia, U.S.A., abstracts*. International Nannoplankton Association, Inc. Harcourt Colour Print, Swansea, U.K. (2013).

18. Kocsis, K., Hyttinen, M., Helminen, H.J., Aydelotte, M.B. & Módis, L. Combination of digital image analysis and polarisation microscopy: theoretical considerations and experimental data. *Microscopy research and technique* **43** 511-517 (1998).

19. Sørensen, B.E. A revised Michel-Lévy interference colour chart based on first-principles calculations *European Journal of Mineralogy* **25** 5-10 (2012).
